# Fluorescent Fe Embedded Magnetic Nanodiamonds Made by Ion Implantation

**DOI:** 10.1038/s41598-018-37820-z

**Published:** 2019-02-04

**Authors:** Bo-Rong Lin, Srinivasu Kunuku, Chien-Hsu Chen, Tzung-Yuang Chen, Tung-Yuan Hsiao, Hung-Kai Yu, Yu-Jen Chang, Li-Chuan Liao, Huan Niu, Chien-Ping Lee

**Affiliations:** 1 Department of Electronics Engineering and Institute of Electronics, National Chiao Tung University, Hsinchu, Taiwan; 2 Accelerator Laboratory, Nuclear Science and Technology Development Center, National Tsing Hua University, Hsinchu, Taiwan; 3 Health Physics Division, Nuclear Science and Technology Development Center, National Tsing Hua University, Hsinchu, Taiwan; 4 Bioresource Collection and Research Center, Food Industry Research and Development Institute, Hsinchu, Taiwan

## Abstract

We demonstrate fluorescent Fe embedded magnetic nanodiamonds by ion implantation and two-step annealing. The diamond characteristics with a highly ordered core and a graphite surface layer are maintained after the implantation process. After the two-step annealing process, a bright red fluorescence associated with nitrogen-vacancy centers is observed. These new fluorescent magnetic nanodiamonds can be used as a dual-function *in vivo* tracer with both optical visibility and magnetic resonance imaging capabilities. They are potentially useful for the more advanced *in vivo* biological and medical applications.

## Introduction

Multi-functional nanoparticles have been utilized in many areas especially medical applications in recent years^1,2^. Among many candidates, nano-diamonds (NDs) have been perceived as the most promising material for bio-applications due to their excellent bio-compatibilities^3,4^ and versatility^5–7^. One of the most attractive features of NDs is their capability to be functionalized by surface modification^8,9^. Designed functional groups can be easily attached to their surfaces chemically enabling them to load drugs and to target specific proteins^10^. However, not all chemicals that are desirable to be attached to the ND surface are nontoxic. One example is iron compounds, which can make NDs magnetic but might be toxic to human bodies. Magnetic NDs are potentially very useful for magnetic resonance imaging (MRI)^11,12^ and targeted radio frequency thermal therapy^13^.

Recently, we have developed a technique to place Fe atoms into the NDs by ion implantation^14^. Because the Fe atoms are embedded inside the NDs and are not in direct contact with the outside world, they are not toxic to living cells and at the same time become magnetic. We have shown that these magnetic NDs can be used as an effective MRI contrast agent without cytotoxicity^15^.

We have found unexpectedly that these Fe implanted magnetic NDs have a strong fluorescence if they are treated properly. It is known that there are nitrogen impurities naturally incorporated in NDs through the fabrication process. When the substitutional nitrogen atoms and adjacent lattice vacancies form complexes, they can give out red fluorescent light. The vacancies can be artificially generated by high energy He ion bombardment^16,17^. Then a thermal treatment is usually used to cause the formation of the nitrogen-vacancy (NV) light emitting centers^16^. The fluorescent NDs are good tracers inside living cells and animal bodies^18,19^. The red fluorescence, with a wavelength of around 700 *nm*, can penetrate deep into biological tissues making it suitable for *in vivo* imaging^20^.

In this work, we report fluorescent Fe embedded magnetic NDs. During the ion implantation processes of our magnetic NDs, the Fe ions penetrate into the diamond lattice creating the magnetism. At the same time, many vacancies are formed because of the high energy ion bombardment. After a high-temperature heat treatment, a very bright red fluorescence is observed. So similar to He-ion bombardment, Fe implantation and subsequent thermal annealing in our process can also generate a lot of nitrogen-vacancy light emitting centers. This enables our NDs to be not only magnetic but also optically visible. Combining optical visibility and MRI capability, the Fe embedded magnetic NDs become a dual-function *in vivo* tracer. In the optically accessible body region, we can use the fluorescence images to trace them. For the region deep in the body, we can use the MR images to capture them. So the fluorescent Fe embedded magnetic NDs will extend the practical use of NDs and open a new biological research field in the future.

## Samples and Experiments

NDs powder (~100 *nm* in diameter, Microdiamant Co.) was first dispersed in deionized water and then coated on an oxidized silicon wafer. Fe-ions were implanted into NDs with an energy of 150 *keV* and a dose of 5 × 10^15^ *ions/cm*^2^. After that, the magnetic NDs were collected^14^ and then spread on a silicon wafer. The NDs were first annealed at 800 °C for 2 hours in vacuum. Following that they were further heat treated in the air at 450 °C for 3 hours. The purpose of such a two-step annealing process will be described in the following.

## Results and Discussion

**Figure 1:**
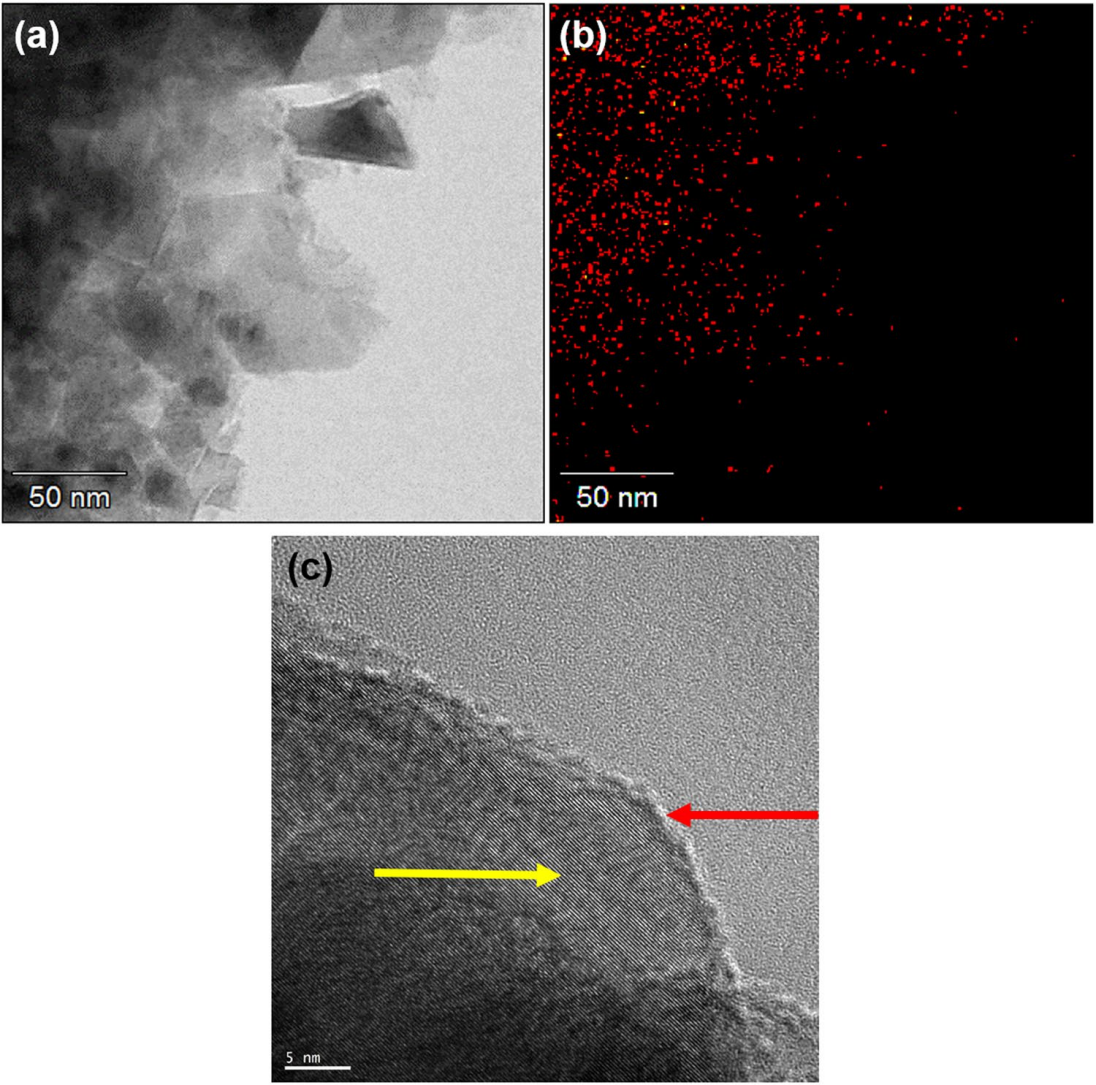
(**a**) TEM image of as-implanted NDs. (**b**) EDS mapping for Fe. Red points represent the position with iron signal. (**c**) High-resolution TEM image of the as-implanted NDs.

After the Fe ion implantation, the first thing that we need to know is where the implanted Fe atoms were located in the NDs. The TEM image of a cluster of NDs after implantation is shown in Fig. 1(a). The corresponding Fe distribution of the same group of NDs measured by energy dispersive spectroscopy (EDS) mapping is shown in Fig. 1(b). We can see from these images that the Fe atoms are distributed well inside the NDs. Figure 1(c) shows a high-resolution TEM image of a Fe-ND. A highly ordered diamond core (yellow arrow) covered by a graphite layer (red arrow) with a few nanometers thickness is clearly seen. This image demonstrates that the implantation process does not change the structural integrity of the NDs. The graphite layer, which has been present in the original NDs and further thickened by the implantation process is undesirable not only because it changes the sp^3^ surface structure but also it can impede the fluorescent emission from the NDs^21,22^. As will be described in the following, this graphite layer can be easily removed through an annealing process.

The luminescence from NDs without any post-processing is barely seen because of the lack of emission centers. The Fe ion implantation, however, generates a lot of carbon vacancies in the NDs lattice. These lattice vacancies are unstable and static at room temperature. They become mobile if they are treated at the high temperature, therefore, the annealing process helps the vacancies to diffuse and to combine with N atoms, which are substituted in carbon lattice in NDs to form NV centers^23^. So following Fe ion implantation, our Fe-NDs were annealed at 800 °C in vacuum for the formation of the NV centers. But as shown in Fig. 1(c), there is a graphite layer on the surface of the NDs. S. Osswald *et al*. have shown that a low- temperature annealing in the air can remove such graphite layer by selective oxidation^24^. Following their method, we have performed a second step annealing process at 450 °C in the air to remove the graphite layer. As will be shown in the following photo-luminescence (PL) spectra, this step is crucial for the luminescent property of the Fe-NDs.

**Figure 2:**
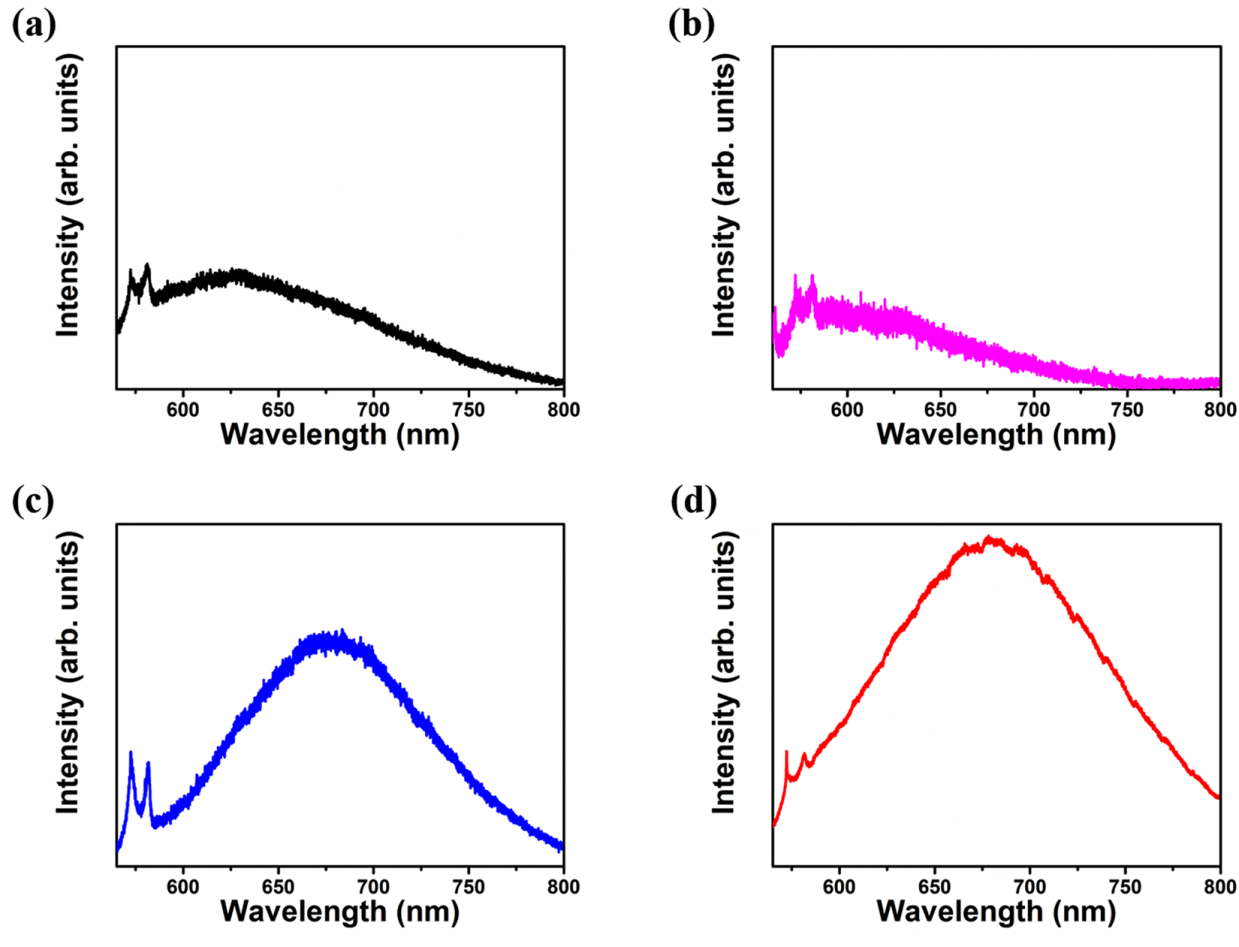
Photoluminescence spectra of (**a**) Original NDs. (**b**) NDs after Fe implantation (**c**) Fe-NDs annealed at 800 °C under vacuum. (**d**) Fe-NDs annealed at 800 °C under vacuum and 450 °C in air.

The fluorescent emission characteristics of NDs and Fe-NDs were studied using the micro-PL measurement system. The pumping source was a diode pumped solid state 532 *nm* laser. Figure 2 shows the PL spectra of NDs and Fe-NDs at different stages of processing. Figure 2(a) shows the PL spectrum of the original NDs prior to Fe implantation. The fluorescent emission of NDs was very weak due to fewer emission centers. The two sharp peaks between 550–600 *nm* are from Raman scattering of diamond and graphite^25–27^. After Fe implantation, the emitted light became even weaker as shown in Fig. 2(b). It was because the diamond lattice was damaged by the high-energy implanted Fe ions. But at the same time, many carbon vacancies were generated inside the NDs. After annealing at 800 °C in the vacuum, the vacancies are started to move to the vicinity of N atoms and captured by the substitutional N atoms in the Fe-NDs^23^. As a result, intense red fluorescence illustrated around 680 *nm* from the 800 °C annealed Fe-NDs (See Fig. 2(c)). A board spectrum shape was observed, which is a typical characteristic of the negatively charged nitrogen-vacancy (NV^−^) center’s emission^28,29^.

Furthermore, second-step annealing was executed at 450 °C in the air in order to remove graphite on the surface of annealed Fe-NDs in first-step, thus the emission of the red fluorescence became very strong (see Fig. 2(d)). The second-step annealing revealed a significant effect on the NV^−^ emission intensity, which appeared in the PL spectrum of two-step annealed Fe-NDs (Fig. 2(d)). The prominent enhancement of NV^−^ emission is due to two aspects. The first one is the annealing at 450 °C in the air is similar to the oxidation of Fe-NDs, such an oxidation influence the change of charge states of NV° to NV^−^ centers^30^. As a result, in the second-step annealing process, most of the NV° centers changed their charge state to NV^−^, consequently, the intensity of red luminescence was increased significantly. The second one is very straightforward. As the graphite on Fe-ND’s surface has been removed by the oxidation process in the air at 450 °C, the quenching effect on NV^−^ emission by graphite is overwhelmingly suppressed^31^. It should be noted that the intensity of the emission from these set of samples cannot be compared directly, since the intensity shown as arbitrary units. But from the signal to noise ratio of these spectra, we can tell that the luminance from the Fe-NDs with two-step annealing is much stronger than that of all others samples.

**Figure 3:**
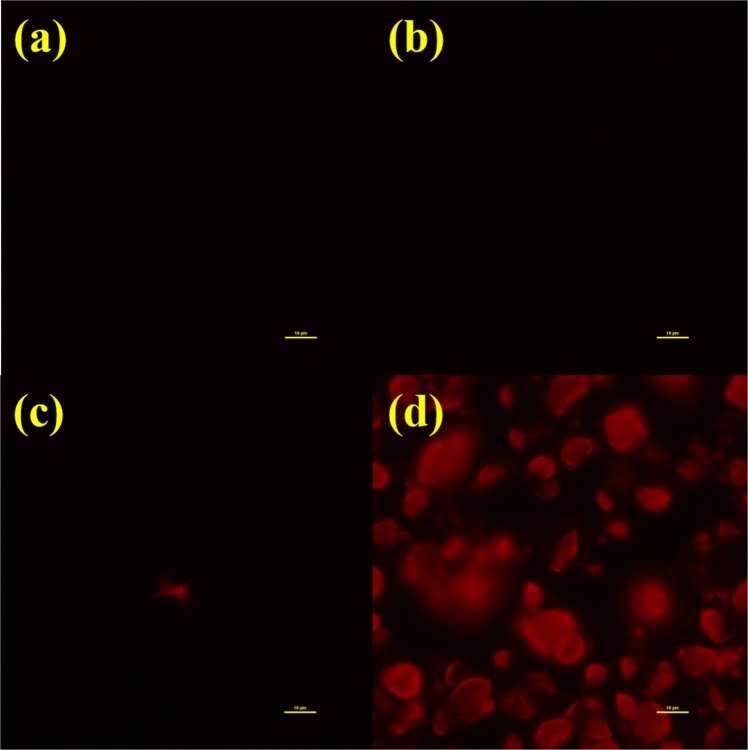
Microscope fluorescence images of NDs and Fe-NDs under different condition. (**a**) Original NDs. (**b**) NDs after Fe implantation (**c**) Fe-NDs annealed at 800 °C under vacuum. (**d**) Fe-NDs annealed at 800 °C under vacuum and 450 °C in the air. Scale bar: 10 *μm*.

**Figure 4:**
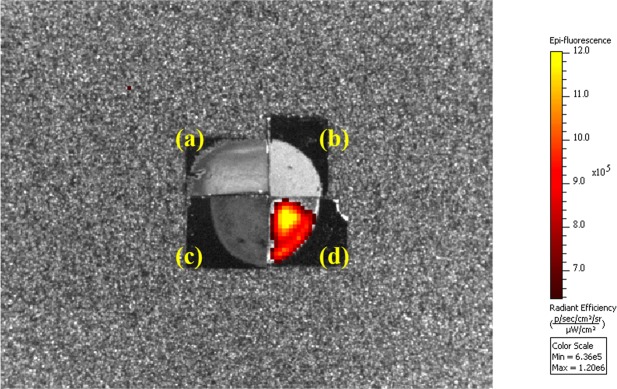
IVIS fluorescence images of NDs and Fe-NDs under different condition. (**a**) Original NDs. (**b**) NDs after Fe implantation (**c**) Fe-NDs annealed at 800 °C under vacuum. (**d**) Fe-NDs annealed at 800 °C under vacuum and 450 °C in the air. The grey image is taken by visible light camera and then the fluorescent signal is overlaid on this grey image. Only the colored part has 680 *nm* fluorescence emission signal. The dark area is uncoated silicon wafer surface.

For the fluorescence to be useful, it has to be observable under real imaging systems. We have used a Nikon inverted fluorescence microscope and an *in vivo* imaging system (IVIS Lumina II) to look at the real images of our NDs and Fe-NDs. The excitation light had a wavelength around 550 *nm* and the detection window was centered around 680 *nm*. Figure 3(a–d) are fluorescent images of NDs and Fe-NDs were captured by the Nikon microscope. The dark images were from, (a) original NDs, (b) Fe-NDs and (c) Fe-NDs annealed at 800 °C in the vacuum. These three cases illustrate that no significant fluorescence from the NV color centers of the NDs, Fe-NDs and 800 °C annealed Fe-NDs. Whereas, a noticeable fluorescence has been perceived from the two-step annealed Fe-NDs, which has shown in Fig. 3(d). The corresponding IVIS fluorescence images are shown in Fig. 4(a–d). These images depict the macroscopic pictures of the NDs (Fe-NDs) covered Si wafers. The grey image is taken by the visible light camera and then the fluorescent signal is overlaid on this grey image. Only the colored part emits the 680 *nm* fluorescence emission signal. Again, the only visible fluorescence was from the one with Fe-NDs after two-step annealing. IVIS is a very mature and sensitive technique commonly used for the biological organism. The fluorescence images are shown here demonstrate that our Fe-NDs are indeed useful as a luminous agent.

It should be mentioned that we have also investigated the PL and fluorescent images at NDs, which did not receive Fe implantation but underwent the same annealing processes as the Fe-NDs. The resultant PL spectra indicated weak NV emission, but it was totally invisible under the fluorescence imaging system. According to our study, both ion implantation and subsequent two-step annealing processes are necessary for the emission of red fluorescent light for bio-imaging applications.

## Conclusion

In conclusion, we have successfully fabricated fluorescent Fe embedded magnetic NDs by Fe ion implantation and two-step annealing. These magnetic NDs have strong red fluorescence that is easily detectable by fluorescence microscopes and IVIS systems. Combining the optical visibility and the magnetic property, fluorescent Fe embedded NDs can be used as a dual-function *in vivo* tracer. This new material should bring new applications for nano-medicine in the future.

## References

[1] Bao G, Mitragotri S, Tong S (2013). Multifunctional nanoparticles for drug delivery and molecular imaging. Annu Rev Biomed Eng.

[2] Srinivasan M, Rajabi M, Mousa SA (2015). Multifunctional Nanomaterials and Their Applications in Drug Delivery and Cancer Therapy. Nanomaterials (Basel).

[3] Narayan RJ (2006). Microstructural and biological properties of nanocrystalline diamond coatings. Diamond and Related Materials.

[4] Moore L (2016). Biocompatibility Assessment of Detonation Nanodiamond in Non-Human Primates and Rats Using Histological, Hematologic, and Urine Analysis. ACS Nano.

[5] Turcheniuk K, Mochalin VN (2017). Biomedical applications of nanodiamond (Review). Nanotechnology.

[6] van der Laan K, Hasani M, Zheng T, Schirhagl R (2018). Nanodiamonds for *In Vivo* Applications. Small.

[7] Chipaux M (2018). Nanodiamonds and Their Applications in Cells. Small.

[8] Krueger A, Stegk J, Liang Y, Lu L, Jarre G (2008). Biotinylated nanodiamond: simple and efficient functionalization of detonation diamond. Langmuir.

[9] Liang Y, Ozawa M, Krueger A (2009). A general procedure to functionalize agglomerating nanoparticles demonstrated on nanodiamond. ACS Nano.

[10] Zhu Y (2012). The biocompatibility of nanodiamonds and their application in drug delivery systems. Theranostics.

[11] Manus LM (2010). Gd(III)-nanodiamond conjugates for MRI contrast enhancement. Nano Lett.

[12] Rammohan N (2016). Nanodiamond-Gadolinium(III) Aggregates for Tracking Cancer Growth *In Vivo* at High Field. Nano Lett.

[13] Lee JH (2011). Exchange-coupled magnetic nanoparticles for efficient heat induction. Nat Nanotechnol.

[14] Chen C, Cho IC, Jian HS, Niu H (2017). Fe doped Magnetic Nanodiamonds made by Ion Implantation. Sci Rep.

[15] Lin BR (2018). Fe Doped Magnetic Nanodiamonds Made by Ion Implantation as Contrast Agent for MRI. Sci Rep.

[16] Chang YR (2008). Mass production and dynamic imaging of fluorescent nanodiamonds. Nat Nanotechnol.

[17] Wu TJ (2013). Tracking the engraftment and regenerative capabilities of transplanted lung stem cells using fluorescent nanodiamonds. Nat Nanotechnol.

[18] Xing Y, Dai L (2009). Nanodiamonds for nanomedicine. Nanomedicine.

[19] Yuan Y (2010). Pulmonary toxicity and translocation of nanodiamonds in mice. Diamond and Related Materials.

[20] Smith AM, Mancini MC, Nie S (2009). Bioimaging: second window for *in vivo* imaging. Nat Nanotechnol.

[21] Smith BR (2009). Five-nanometer diamond with luminescent nitrogen-vacancy defect centers. Small.

[22] Bradac C (2010). Observation and control of blinking nitrogen-vacancy centres in discrete nanodiamonds. Nat Nanotechnol.

[23] Haque A, Sumaiya S (2017). An Overview on the Formation and Processing of Nitrogen-Vacancy Photonic Centers in Diamond by Ion Implantation. Journal of Manufacturing and Materials Processing.

[24] Osswald S, Yushin G, Mochalin V, Kucheyev SO, Gogotsi Y (2006). Control of sp2/sp3 Carbon Ratio and Surface Chemistry of Nanodiamond Powders by Selective Oxidation in Air. Journal of the American Chemical Society.

[25] Kurtsiefer C, Mayer S, Zarda P, Weinfurter H (2000). Stable Solid-State Source of Single Photons. Physical Review Letters.

[26] Vlasov II (2010). Nitrogen and luminescent nitrogen-vacancy defects in detonation nanodiamond. Small.

[27] Sébastien P, Detlef R, Dominik W, Jan M, Alexander Z (2011). Creation and nature of optical centres in diamond for single-photon emission—overview and critical remarks. New Journal of Physics.

[28] Aharonovich I (2011). Diamond-based single-photon emitters. Reports on Progress in Physics.

[29] Zaitsev, A. M. *Optical properties of diamond: a data handbook*. (Springer Science & Business Media, 2013).

[30] Fu KMC, Santori C, Barclay PE, Beausoleil RG (2010). Conversion of neutral nitrogen-vacancy centers to negatively charged nitrogen-vacancy centers through selective oxidation. Applied Physics Letters.

[31] Smith BR, Gruber D, Plakhotnik T (2010). The effects of surface oxidation on luminescence of nano diamonds. Diamond and Related Materials.

